# Association between tidal volume and mortality in patients without acute respiratory distress syndrome: A systematic review and meta-analysis

**DOI:** 10.3892/br.2026.2171

**Published:** 2026-06-23

**Authors:** Mingyuan Gu, Feiping Xia, Zhonghua Lu, Shanshan Meng, Fengmei Guo

**Affiliations:** Department of Critical Care Medicine, Zhongda Hospital, School of Medicine, Southeast University, Nanjing, Jiangsu 210009, P.R. China

**Keywords:** low tidal volume, mortality, Pmax, patients without acute respiratory distress syndrome, meta-analysis

## Abstract

The optimal tidal volume for mechanically ventilated patients without acute respiratory distress syndrome (ARDS) remains unclear. The present systematic review and meta-analysis compared the effects of low compared with high tidal volume (Vt) ventilation in non-ARDS patients. Randomized controlled trials and observational studies comparing low and high Vt in adults without ARDS were included. The primary outcome was short-term mortality (28- or 30-day mortality in the ICU or hospital). Secondary outcomes were days of ventilation, pulmonary complications and length of hospital stay. Summary odds ratios (ORs) and 95% confidence intervals (CIs) were calculated using a random-effects model. In total, 18 studies were included (1,530 low Vt vs. 1,512 high Vt patients). No significant difference in short term mortality was observed between low and high Vt ventilation (n=12; OR, 0.88; 95% CI, 0.71-1.09; P=0.24). However, subgroup analysis by maximum airway pressure (Pmax) showed that low Vt with low Pmax significantly reduced short-term mortality compared with low Vt with Pmax (OR, 0.50; 95% CI, 0.34-0.75; P=0.0007). Low Vt also decreased days of ventilation [mean difference (MD), -1.00; 95% CI, -1.76--0.24; P=0.01], pulmonary complications (n=11 studies; OR, 0.40; 95% CI, 0.30-0.54; P<0.00001) and hospital stay (n=11 studies; MD, -1.49; 95% CI, -2.54--0.44; P=0.006). Trial sequential analysis indicated conclusive evidence, suggesting further trials are unlikely to change the conclusion. In conclusion, low Vt combined with low Pmax, but not low Vt alone, improved short-term mortality in non-ARDS patients with respiratory failure. Clinically, this suggests that limiting both tidal volume and airway pressure may offer a protective ventilation strategy for non-ARDS patients, potentially guiding bedside titration of ventilatory settings to reduce mortality. The present study was registered in PROSPERO (https://www.crd.york.ac.uk/PROSPERO/view/CRD42019119453).

## Introduction

Mechanical ventilation is the standard treatment for patients with acute respiratory distress syndrome (ARDS). However, a high tidal volume (Vt) may overstretch alveoli, cause ventilator-associated lung injury (1) and increase mortality (2), partly through proinflammatory mediator release induced by biotrauma and volutrauma (3). In ARDS patients, lung-protective ventilation with low Vt has been firmly established as the standard of care based on landmark trials.

By contrast, the protective effect of low Vt ventilation in patients who received mechanical ventilation but do not have ARDS remains controversial. Driving pressure (∆P) is also important for the optimization of mechanical ventilation parameters. Unlike the well-defined benefits in ARDS, the optimal Vt strategy in non-ARDS patients is less clear, as these patients have relatively healthy lungs and may be more tolerant of higher Vt, but are also potentially at risk of developing ventilator-induced lung injury. Certain studies have reported increased morbidity and mortality with high Vt in non-ARDS patients (4,5) and meta-analyses have demonstrated reduced mortality, ventilation duration (6) and pulmonary complications (7) with low Vt in non-ARDS patients. Conversely, a previous randomized control trial (RCT) reported no mortality or ventilation benefit of low Vt compared with that of intermediate Vt in patients without ARDS (8). These conflicting findings highlight a knowledge gap that may require large RCTs to resolve (9).

To address this gap, the present study performed a meta-analysis evaluating low compared with high Vt in patients without ARDS. As outcomes are also influenced by positive endexpiratory pressure (PEEP) (10), ΔP (11) and maximum airway pressure (Pmax) (12), subgroup analyses stratified by these parameters were performed. Additionally, trial sequential analysis (TSA) was applied to assess the robustness and conclusiveness of the evidence. Thus, the present study not only compared low vs. high Vt in non-ARDS patients, but also explored whether confounding factors such as Pmax, PEEP and ΔP modified the relationship between Vt and clinical outcomes, providing a more comprehensive evidence base for bedside ventilation management.

## Materials and methods

### Study design and registration

The present systematic review and meta-analysis was performed according to the Preferred Reporting Items for Systematic Reviews and Meta-analyses (PRISMA) guidelines (13) and the recommendations of the Meta-analysis of Observational Studies in Epidemiology Group (14).

### Data sources and search strategies

The PubMed, Cochrane Library and EMBASE databases were searched from inception to February 2025 using the following Medical Subject Headings and keywords (‘low Vt’ OR ‘protective ventilation’ OR ‘lower Vt’). No limits were applied for language. The reference lists of eligible studies were also searched to identify additional relevant articles.

### Inclusion and exclusion criteria

The inclusion criteria were as follows: i) RCTs or observational studies that reported Vt; ii) patients without ARDS at the start of ventilation (patients without ARDS were defined as mechanically ventilated adults who did not meet the Berlin definition criteria for ARDS (15), consistent with the inclusion criteria of the major trials included in this meta-analysis) (8); and iii) adult patients (aged ≥18 years) undergoing uninterrupted ventilation. The exclusion criteria were as follows: i) Patients who underwent one-lung ventilation; ii) patients with ARDS at the start of ventilation; and iii) reviews, letters, abstracts or editorials. RCTs and observational studies were both included as observational studies can provide real-world evidence and larger sample sizes when RCTs are limited; a sensitivity analysis was employed to assess the effect of including observational studies on the overall findings.

### Study selection

A total of two reviewers (MG and FX) independently assessed titles and abstracts to select eligible studies. Full text records were retrieved and reviewed to determine study eligibility. Reviewers were blinded to authors' names, year of publication and journal. Disagreements regarding study selection were resolved by discussion and consensus.

### Data extraction and study endpoints

The reviewers independently extracted data from eligible studies, including the author, sample size, study design, patient history of chronic pulmonary disease, study limitations and outcome measures.

The primary outcome was short-term mortality, defined as 28- or 30-day mortality in the intensive care unit (ICU) or hospital. Secondary outcomes were the number of days of ventilation, incidence of pulmonary complications and length of hospital stay.

### Assessment of the quality of evidence in included studies

The reviewers independently assessed the methodological quality and risk of bias of each included study. RCTs were assessed using the Cochrane risk of bias tool (16), cohort studies were assessed using the Newcastle-Ottawa scale and cross-sectional studies were assessed using the Agency for Healthcare Research and Quality methodology checklist (17,18). The presence of publication bias was evaluated by visual inspection of funnel plots (19). The strength of evidence was determined using GRADE (20). Disagreements regarding quality of evidence were resolved by discussion and consensus.

### Data synthesis and statistical analysis

Statistical analysis was performed using RevMan (v. 5.3; The Cochrane Collaboration) and STATA (v. 12.0; StataCorp LP) software. Odds ratios (ORs) and corresponding 95% confidence intervals (CIs) were calculated using the inverse-variance weighted approach. Heterogeneity between studies was assessed using the I^2^ statistic (I^2^ ≥50% indicating substantial heterogeneity).

Low Vt was defined as <8 ml/kg predicted body weight, and high Vt was defined as >9 ml/kg of predicted body weight. The effects of PEEP, ∆P and Pmax were assessed as confounding factors. The subgroup analysis stratified by Pmax was prespecified in the PROSPERO registration (registration no. CRD42019119453). The following subgroup analyses were predefined: Stratification by PEEP, ΔP and Pmax, based on their known influence on outcomes in mechanically ventilated patients. Specifically, Pmax was categorized as low (≤25 cmH_2_O) or high (>25 cmH_2_O) based on previously published thresholds (6,7). Pmax was defined as peak airway pressure in pressure assist-control ventilation and plateau pressure in volume assist-control ventilation. Although peak pressure and plateau pressure are physiologically distinct, both are indicators of Pmax exposure, and limited primary studies reported both values separately. Furthermore, in pressure-controlled ventilation modes commonly used in intraoperative settings, peak pressure approximates plateau pressure in the absence of significant airway resistance (4,10). Given the exploratory nature of this subgroup analysis, studies were pooled using either definition while acknowledging this as a limitation. Data describing PEEP, ∆P and Pmax were extracted, when available. A meta-regression analysis was performed to evaluate the associations between low vs. high Vt and PEEP, ∆P and Pmax. Subgroup analysis was stratified by low Vt and low compared with high Pmax. TSA was applied to control the risk of type I and type II errors due to sparse data and repeated significance testing. TSA calculated the required information size and monitored whether the cumulative evidence crossed the monitoring boundaries, thereby assessing the conclusiveness of the findings (21). P<0.05 was considered to indicate a statistically significant difference.

## Results

### Study characteristics

**Figure 1:**
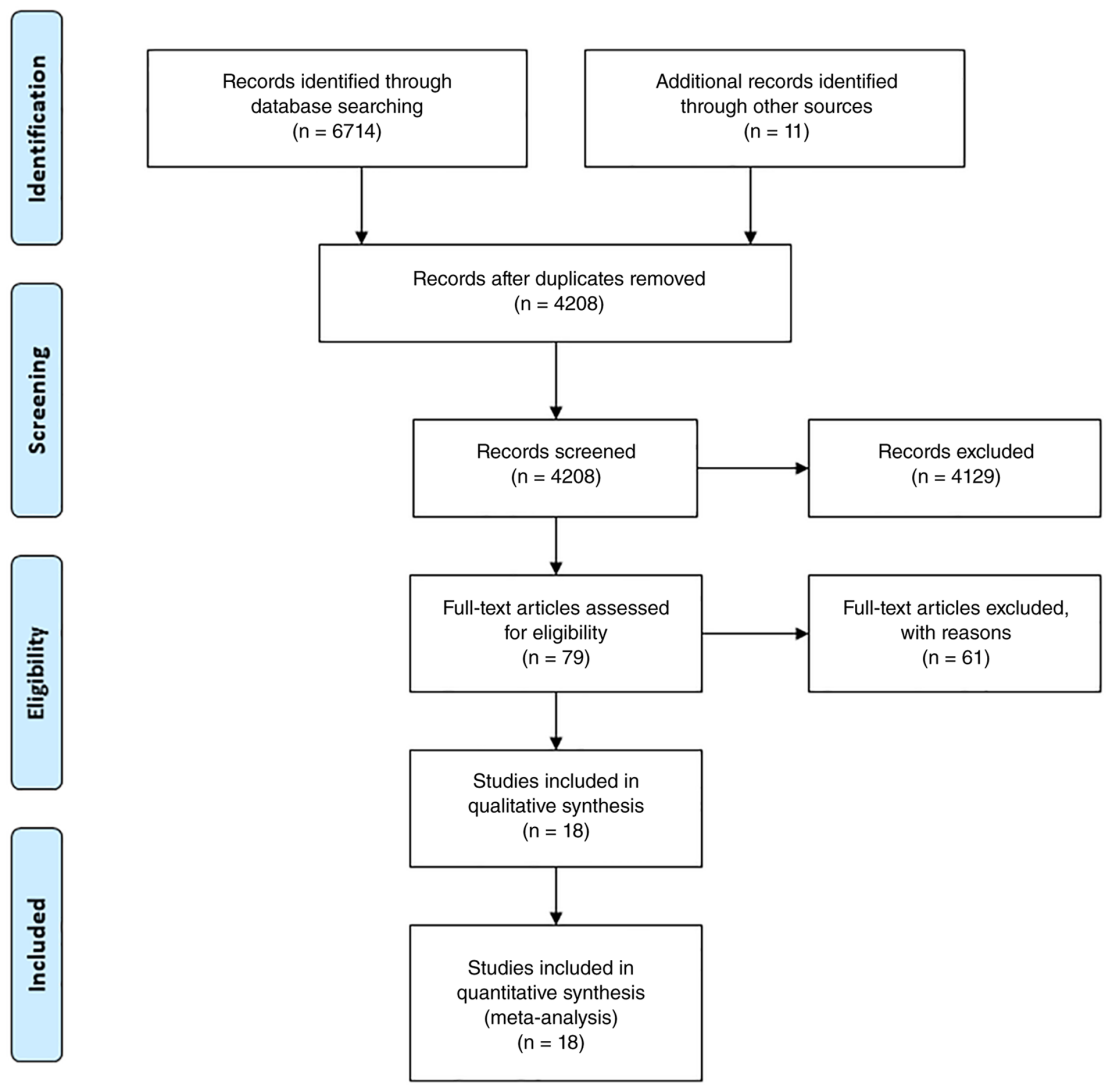
Preferred reporting items for systematic reviews and meta-analyses flow diagram of study selection.

The searches identified 6,725 citations, and the full text articles of 79 studies were reviewed. Finally, 18 studies (8,22-38), comprising 2 observational studies (23,37) and 16 RCTs, were included in the meta-analysis (Fig. 1). The characteristics of the included studies are presented in Table I. A total of 3,042 adult patients [low Vt, 1,530 (50.3%); high Vt, 1,512 (49.7%)] were included in the analyses. Of these, 11 studies were ICU-based and 7 were intraoperative; the mixture of these two settings may have increased clinical heterogeneity and this represents a limitation of the meta-analysis. The two settings were included as they represent the two main populations of mechanically ventilated patients without ARDS, and inclusion was restricted to those meeting the same eligibility criteria. Although the mixture of ICU and intraoperative studies may introduce clinical heterogeneity, consistent inclusion and exclusion criteria were applied across both settings to maximize patient homogeneity. Moreover, subgroup analyses and meta-regression were performed to explore potential sources of heterogeneity.

The risk of bias assessment for RCTs is presented in Fig. S1. Among the 16 RCTs, nine trials were assessed as high risk of performance bias and detection bias (8,25-29,32,34,37) and two trials were assessed as unclear risk of performance bias and detection bias as the authors considered that the outcomes were not inﬂuenced by a lack of blinding or that blinding of participants was impossible (30,31). Trials were assessed as low risk of all other biases. The high risk of bias in these nine trials may influence the reliability of the findings; however, these trials represent the best available evidence given the inherent challenge of blinding ventilator settings. Future trials with blinded outcome assessment are warranted to confirm these results. The cohort study (37) scored nine stars (Table SI) and the cross-sectional study (23) scored seven (Table SII) according to Newcastle-Ottawa scale and Agency for Healthcare Research and Quality methodology checklist separately (17,18).

### Low Vt ventilation does not decrease short-term mortality in patients without ARDS

**Figure 2:**
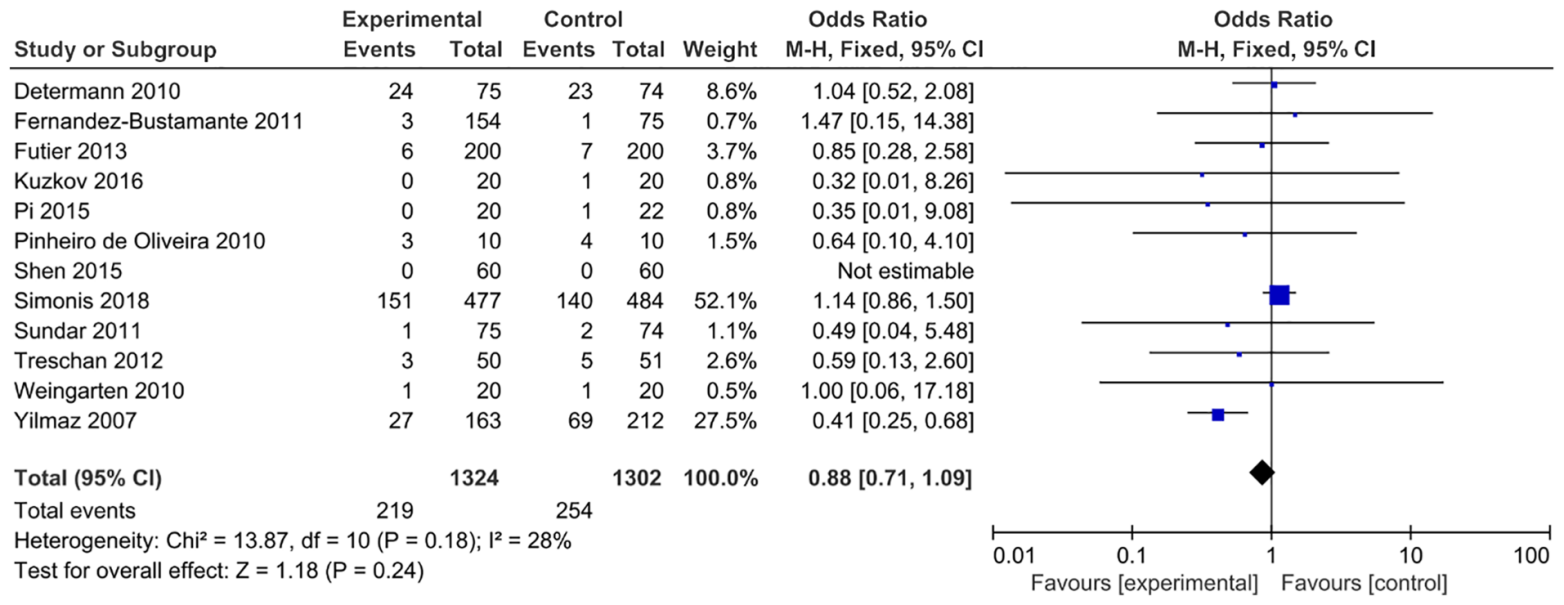
Primary analysis of the effect of ventilation with a low or high Vt on short-term mortality in patients without ARDS. Each square represents the effect size of an individual study, with the size of the square reflecting the study weight. Horizontal lines indicate 95%CI. The diamond at the bottom represents the pooled effect estimate. Vt, tidal volume; ARDS, acute respiratory distress syndrome; CI, confidence intervals; M-H, Mantel-Haenszel.

Short-term mortality was reported in 12 studies (8,22-24,26,29,30,32,34-37) (patients n=2,620). The short-term mortality rate was 16.4% (219/1,324) in patients ventilated with a low Vt and 19.5% (254/1,302) in patients ventilated with a high Vt. The meta-analysis revealed no significant difference in short term mortality between patients ventilated with a low Vt and those ventilated with a high Vt (OR, 0.88; 95% CI, 0.71-1.09; P=0.24; Fig. 2). Thus, low Vt alone did not reduce short-term mortality in patients without ARDS. Moreover, there was no evidence of publication bias (Fig. S2), but the overall strength of evidence was low (Table SIII), suggesting that further investigation may influence the estimate.

### Low Vt and low Pmax decreases short-term mortality in patients without ARDS

**Figure 3:**
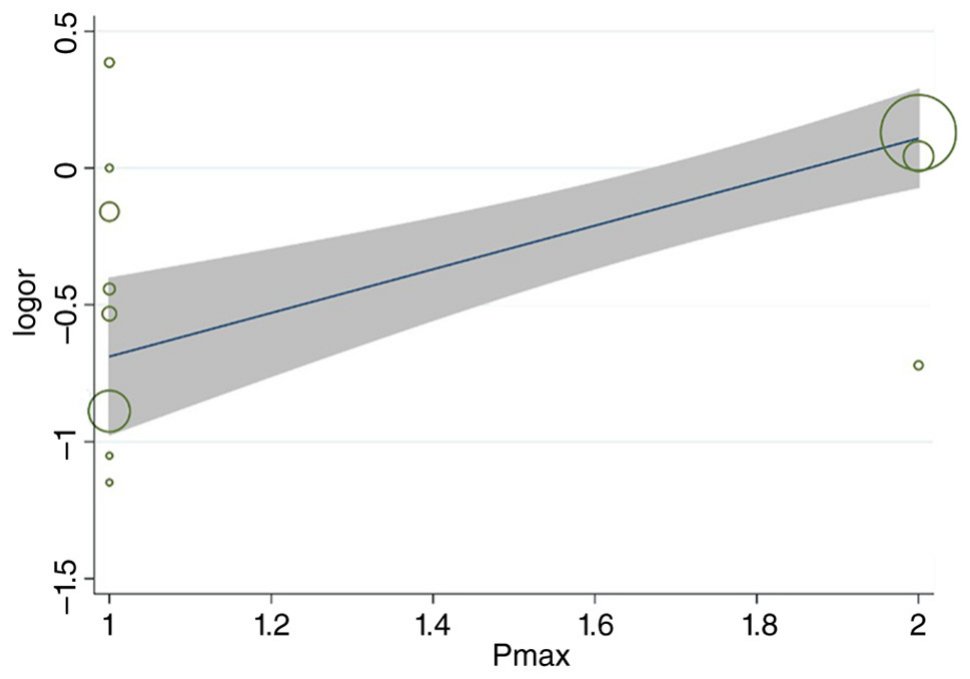
Meta-regression analysis exploring Pmax as a potential confounding factor. Pmax, maximum airway pressure.

**Figure 4:**
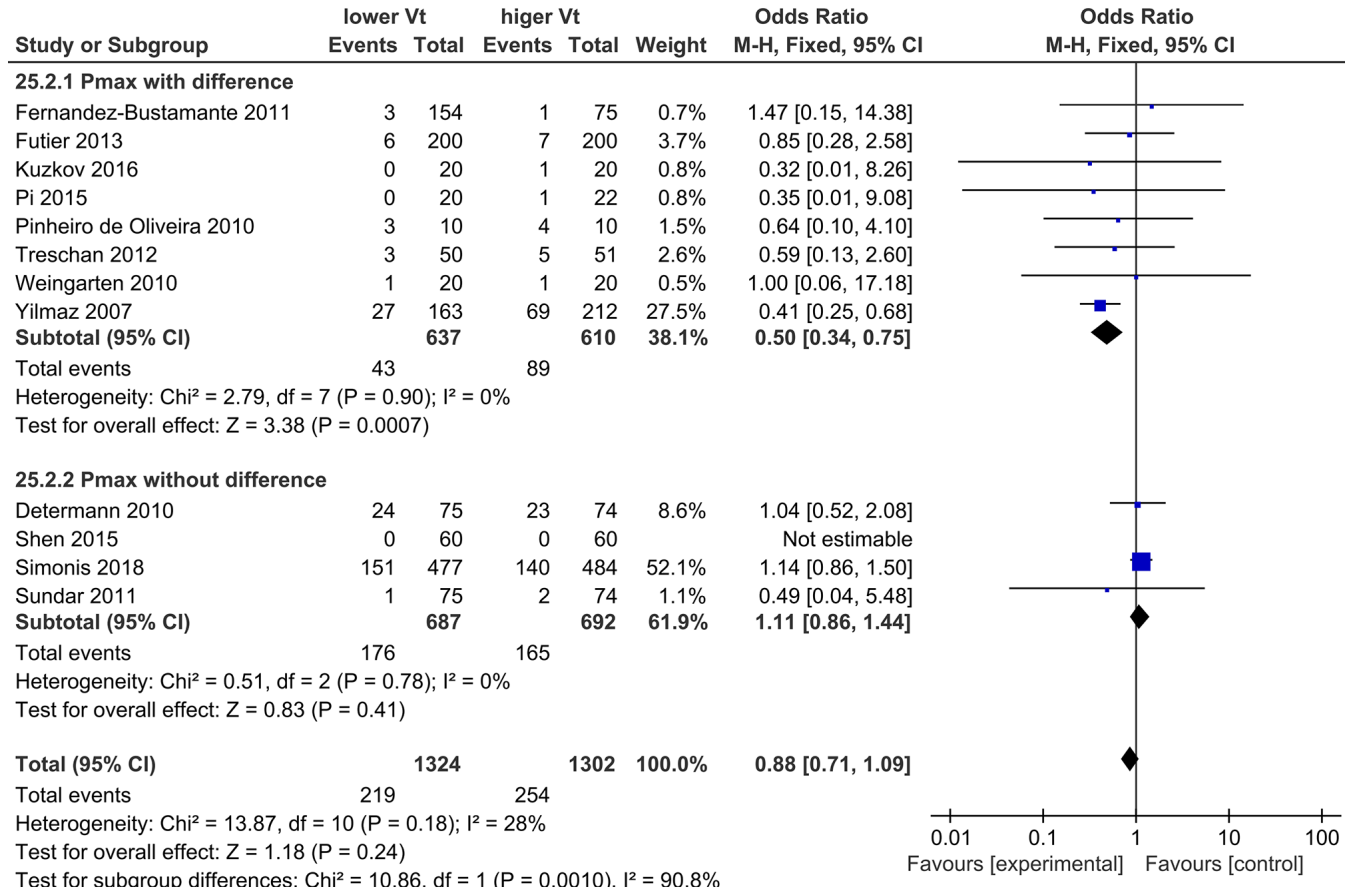
Subgroup analysis of the effect of ventilation with a low Vt and a low or high Pmax on short-term mortality in patients without ARDS. Studies were stratified by Pmax level (low ≤25 cmH_2_O vs. high >25 cmH_2_O). The pooled odds ratio for each subgroup is shown as a diamond, and the overall effect across subgroups is also displayed. Vt, tidal volume; Pmax, maximum airway pressure; ARDS, acute respiratory distress syndrome; CI, confidence intervals; M-H, Mantel-Haenszel.

Meta-regression analyses identified Pmax (P=0.01) as a confounding factor in the analysis of short-term mortality (Figs. 3 and S3). Subgroup analysis stratified by Pmax level in patients receiving low Vt ventilation demonstrated that short-term mortality was significantly decreased in those with low Pmax compared with those with high Pmax (OR, 0.50; 95% CI, 0.34-0.75; P=0.0007; Fig. 4). There was no significant difference in short term mortality between patients ventilated with a low Vt and those ventilated with a high Vt in studies with the same or an undefined Pmax (OR, 1.11; 95% CI, 0.86-1.44; P=0.41; Fig. 4). These findings suggested that a low Vt combined with an appropriate Pmax may improve the outcomes of ventilated patients without ARDS.

**Figure 5:**
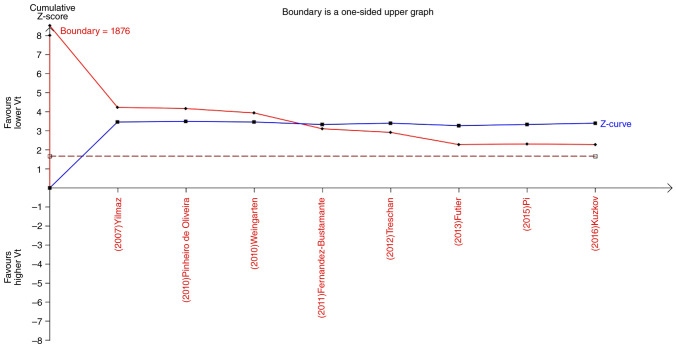
Trial sequential analysis plot for the primary outcome measure (one-sided boundary) of the subgroup analysis. Minimum desired effect size, 16%; statistical power, 80%; control event proportion, 33%; measure of diversity, 0%; observed diversity, 0%.

TSA revealed that the diversity-adjusted required information size was 1,876. The cumulative z-curve crossed the trial sequential monitoring boundary for benefit and the conventional boundary for benefit, but did not reach the estimated required information size (Fig. 5). These findings should be interpreted with caution, as the required information size was not reached. Nevertheless, the crossing of the monitoring boundary suggests a potential benefit that warrants confirmation in future larger trials.

### Low Vt ventilation decreases the number of days of ventilation, incidence of pulmonary complications and hospital length of stay in patients without ARDS

The number of days of ventilation [mean difference (MD), -1.00; 95% CI, -1.76--0.24; P=0.01; Fig. S4], the incidence of pulmonary complications (studies, n=11; OR, 0.40; 95% CI, 0.30-0.54; P<0.00001; Fig. S5) (24-26,28,29,31-33,35,36,38) and hospital length of stay (studies, n=11; MD, -1.49; 95% CI, -2.54--0.44; P=0.006; Fig. S6) (8,23,24,26,28,29,32-36) were significantly decreased in patients ventilated with a low Vt compared with a high Vt. There was evidence of publication bias among studies reporting the number of days of ventilation.

## Discussion

The present systematic review and meta-analysis evaluated the effects of low compared with high Vt ventilation in patients without ARDS. A ventilation strategy combining low Vt with low Pmax was associated with a significant reduction in short-term mortality compared with low Vt combined with high Pmax. Additionally, low Vt ventilation was associated with a reduced duration of ventilation, incidence of pulmonary complications and length of hospital stay compared with high Vt.

The results of the present study demonstrated that low Vt ventilation was not associated with a reduction in short-term mortality in patients without ARDS. Similar to the findings from our previous study (8), the PRoVENT trial reported no significant difference in 28- or 90-day mortality in ICU patients without ARDS who were expected not to be extubated within 24 h of randomization and were ventilated with a low vs. intermediate Vt strategy (39). By contrast, a meta-analysis of 20 articles reported that ventilation with lower Vt was associated with reduced mortality in patients without ARDS (40).

Potentially modifiable factors other than Vt may affect outcomes in patients both with and without ARDS. Decreases in ΔP resulting from changes in ventilator settings have been reported to be strongly associated with increased survival in patients with ARDS (41). ΔP and plateau pressure were risk factors for mortality and ARDS in one cohort study of mechanically ventilated patients without ARDS (11), while ΔP was not associated with hospital mortality in another cohort study of patients without ARDS (42). A secondary analysis of the PRoVENT study reported that a higher Pmax was independently associated with higher in-hospital mortality in critically ill patients under mechanical ventilatory support for reasons other than ARDS (12). Accordingly, in the present meta-analysis, the effects of PEEP, ∆P and Pmax were assessed as confounding factors in short term mortality among ventilated patients without ARDS using meta-regression. Results identified Pmax as a confounding factor and pooled data showed that a ventilation strategy including a low Vt and a low Pmax was associated with a significant reduction in short-term mortality compared with low Vt combined with high Pmax. However, the TSA results should be interpreted with caution, as the required information size was not reached. Nevertheless, the cumulative Z-curve crossed the monitoring boundary for benefit, suggesting a potential survival advantage of low Vt combined with low Pmax that warrants confirmation in future larger trials. Notably, pooled data demonstrated that a ventilation strategy combining low Vt with low Pmax was associated with a significant reduction in short-term mortality compared with low Vt with high Pmax. This finding highlights that limiting both Vt and airway pressure is likely necessary to achieve a survival benefit in patients without ARDS, whereas low Vt alone is insufficient. Ventilation strategies that use a high Vt and a high Pmax may lead to overstretching of alveoli, local production and release of inflammatory mediators, recruitment of neutrophils and ventilator-associated lung injury (1-3).

The findings from the meta-analysis in the present study suggested that duration of ventilation, incidence of pulmonary complications and length of hospital stay may be decreased in patients without ARDS who are ventilated with a low Vt compared with a high Vt. In a post-hoc analysis of a large, randomized trial of low Vt ventilation it was found that during laparoscopic surgeries, low Vt was associated with a markedly reduced pulmonary complications (43). The effect of a low Vt on duration of ventilation is consistent with a previous individual patient data meta-analysis which reported that use of low Vt (≤6 ml/kg of predicted body weight) vs. high Vt (≥10 ml/kg of predicted body weight) in patients without ARDS at the onset of mechanical ventilation was associated with shorter duration of ventilation (6). By contrast, a previously published RCT (8) indicated that a low Vt ventilation strategy was not more effective than an intermediate Vt strategy for decreasing the number of ventilator-free days in patients in the ICU without ARDS. The findings in the present study regarding the effect of a low Vt on the incidence of pulmonary complications and length of hospital stay are consistent with two previous studies (7,40) but contrast with the findings from a third study (8). Consequently, more adequately powered RCTs are needed to evaluate the effect of a low Vt on duration of ventilation, incidence of pulmonary complications and length of hospital stay in patients without ARDS.

Moreover, recent studies support the findings of the present study. For example, one RCT reported that low Vt alone provided no clinical benefit in patients without ARDS (44) and two network meta-analyses both demonstrated that low Vt combined with individualized PEEP reduces pulmonary complications (45,46), which is consistent with the conclusion in the present study that a combination strategy, rather than low Vt alone, is required to improve outcomes.

However, the present meta-analysis has several limitations, including substantial heterogeneity among the included studies; therefore, the results should be interpreted with caution. Specifically, the included studies comprised both ICU-based (11 studies) and intraoperative (seven studies) settings. This mixture may have introduced clinical heterogeneity, as the baseline characteristics, duration of ventilation and severity of illness differ between these two populations. First, data from RCTs and observational studies were pooled; however, the observational studies were of high methodological quality. Second, Pmax varied between studies, and the Pmax setting required for protective mechanical ventilation in patients without ARDS has not been defined. Third, the duration of mechanical ventilation varied across studies. Fourth, Pmax was defined as either peak or plateau pressure across the included studies, and these two variables are physiologically distinct. This heterogeneity in definition may have influenced the results of the Pmax subgroup analysis, and therefore the findings should be interpreted with caution.

In summary, the results of the present study demonstrated that a ventilation strategy combining low Vt with low Pmax was associated with a significant reduction in short-term mortality in patients without ARDS compared with a ventilation strategy combining low Vt with high Pmax. Furthermore, ventilation with a low Vt was associated with a reduced duration of ventilation, incidence of pulmonary complications and length of hospital stay compared with ventilation with a high Vt. Ventilation with a low Vt alone also did not improve short-term mortality. Moreover, the present meta-analysis was limited by heterogeneity between studies.

## Supplementary Material

Risk of bias for included randomized control trials. Red (-) indicates a high risk of bias; yellow (?) indicates unclear risk, and green (+) indicates a low risk of bias.

Funnel plot for the effect of low Vt on short-term mortality in patients without ARDS. Vt, tidal volume; ARDS, acute respiratory distress syndrome.

Meta-regression analyses exploring PEEP and ΔP as potential sources of heterogeneity. (A) Meta-regression analyses of PEEP. (B) Meta-regression analyses of ΔP. PEEP, positive endexpiratory pressure; ΔP, driving pressure.

Number of days of ventilation in patients without ARDS. ARDS, acute respiratory distress syndrome.

Incidence of pulmonary complications in patients without ARDS. ARDS, acute respiratory distress syndrome; M-H, Mantel-Haenszel.

Hospital length of stay in patients without ARDS. ARDS, acute respiratory distress syndrome. CI, confidence intervals.

Newcastle-Ottawa quality assessment scale.

Cross-sectional study quality assessment.

GRADE evidence profile for the studies in the meta-analysis.

## Figures and Tables

**Table I:** Characteristics of the included studies.

			Low Vt			High Vt				
First author/s, year	No.	Cause of ventilation	PEEP (cmH_2_O)	ΔP (cmH_2_O)	Pmax (cmH_2_O)	PEEP (cmH_2_O)	ΔP (cmH_2_O)	Pmax (cmH_2_O)	Main outcome	(Refs.)
Determann *et al*, 2010	150	ICU patients	7	-	-	7	-	-	Cytokine levels in BLF and plasma	(22)
Fernandez-Bustamante *et al*, 2011	429	Abdominal surgery	7	-	20.9	7	-	23.6	Tidal volume setting	(23)
Futier *et al*, 2013	400	Abdominal surgery	6-8	9.2	15.2	0	16.6	16.1	Pulmonary and extrapulmonary complications	(24)
Ge *et al*, 2013	60	Spinal fusion	10	-	-	7	-	-	Pulmonary Complication	(25)
Kuzkov *et al*, 2016	40	Pancreatoduodenal surgery	4	4.6	12.0	4	5.4	16.4	Oxygenation and the incidence of atelectases	(26)
Lee *et al*, 1990	103	Surgery	7	-	28.9	7	-	38.8	The incidence of pulmonary infection	(27)
Park *et al*, 2016	40	Laparoscopic surgery	5	-	-	0	-	-	Pulmonary complications	(28)
Pi *et al*, 2015	63	Abdominal surgery	9	6.7	12.0	0	10.9	16.4	PaO_2_/FiO_2_ ratio and pulmonary compliance	(29)
Pinheiro de Oliveira *et al*, 2010	20	Scheduled surgery	5	12.2	17.9	5	24.3	29.8	Pulmonary inflammation	(30)
Severgnini *et al*, 2013	53	Abdominal surgery	10	8.7	-	0	16.0	-	Pulmonary Infection Score	(31)
Shen *et al*, 2015	120	Surgery	6	-	-	0	-	-	Pulmonary compliance	(32)
Simonis *et al*, 2018	961	ICU patients	7	11.2	-	7	13.0	-	Ventilator-free days	(8)
Soh *et al*, 2018	78	Spinal surgery	6	9.5	16.4	0	15.2	16.3	Pulmonary complications	(33)
Sundar *et al*, 2011	149	Cardiac surgery	5-6	13.9	-	5-6	15.2	-	Time to extubation.	(34)
Treschan *et al*, 2012	101	Abdominal surgery	5	-	15.0	5	-	17.0	Lung function	(35)
Weingarten *et al*, 2010	40	Abdominal surgery	12	-	14.6	0	-	16.8	Oxygenation, respiratory system mechanics	(36)
Yilmaz *et al*, 2007	375	ICU patients	5	-	25.0	5	-	30.9	The frequency of acute lung injury	(37)
Zamani *et al*, 2017	61	CABG surgery	10	-	-	0	-	-	Postoperative pulmonary complications	(38)

Vt, tidal volume (ml/kg predicted body weight); CABG, coronary artery bypass grafting; BLF, bronchoalveolar lavage fluid; ∆P, driving pressure; Pmax, maximum airway pressure.

## Data Availability

The data generated in the present study are included in the figures and/or tables of this article.
