# Supplementary material for: Association between tidal volume and mortality in patients without acute respiratory distress syndrome: A systematic review and meta-analysis
Source: Biomed Rep. 2026 Jun 23;25(2):98. doi: 10.3892/br.2026.2171 (PMC13320072; doi:10.3892/br.2026.2171)

Figure S1. Risk of bias for included randomized control trials. Red (-) indicates a high risk of bias; yellow (?) indicates unclear risk, and green (+) indicates a low risk of bias.

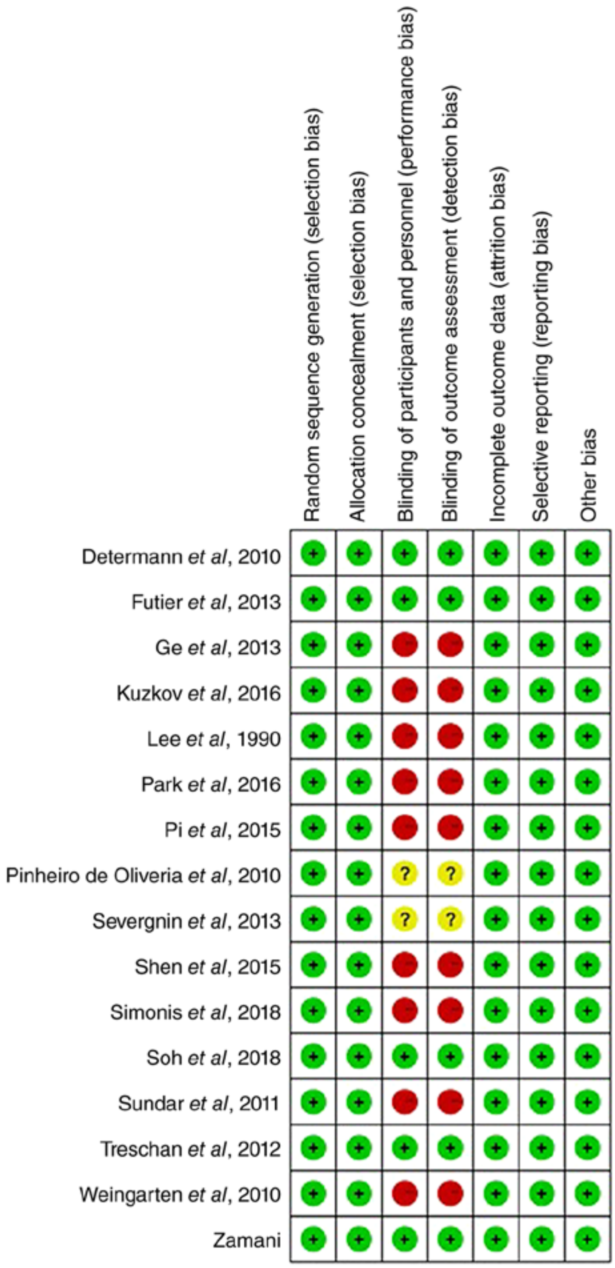

Figure S2. Funnel plot for the effect of low Vt on short-term mortality in patients without ARDS. Vt, tidal volume; ARDS, acute respiratory distress syndrome.

Funnel plot for meta-analysis of the effect of lower tidal volume on short-term mortality in patients without ARDS. Plots were symmetrically distributed, indicating no obvious publication bias. Each point represents one trial.

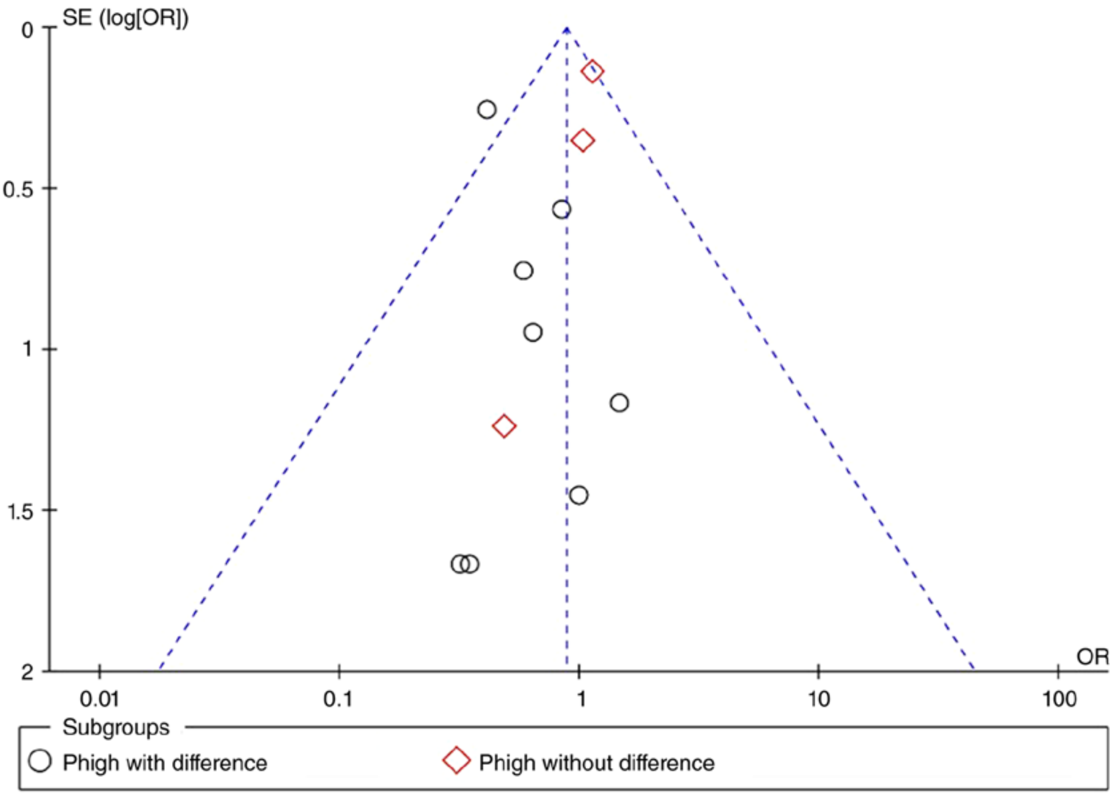

Figure S3. Meta-regression analyses exploring PEEP and ΔP as potential sources of heterogeneity. (A) Meta-regression analyses of PEEP. (B) Meta-regression analyses of ΔP. PEEP, positive endexpiratory pressure; ΔP, driving pressure.

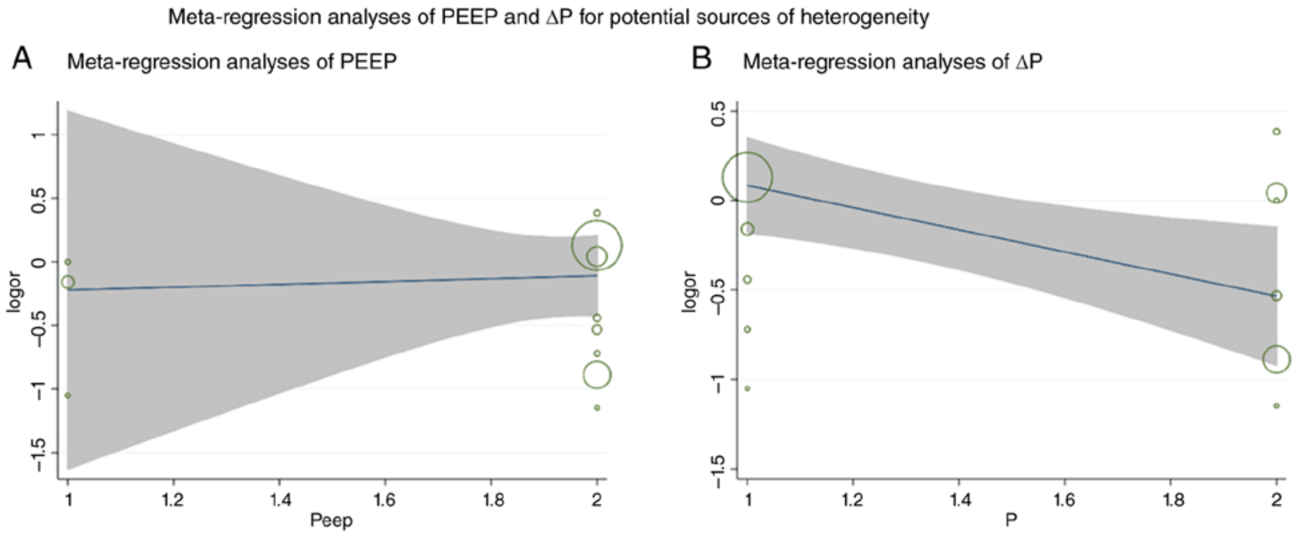

Figure S4. Number of days of ventilation in patients without ARDS. ARDS, acute respiratory distress syndrome.

Days of ventilation in patients without ARDS available for lower versus higher tidal volume. IV = Inverse variance.

	Lower Vt			Higher Vt				Mean Difference
**Study or Subgroup**	**Mean**	**SD**	**Total**	**Mean**	**SD**	**Total**	**Weight**	**IV, Random, 95% CI**
Fernandez-Bustamante 2011	1.5	2.8	154	2.1	3.7	75	20.2%	-0.60 [-1.55, 0.35]
Lee 1990	2.3	0.5	47	3.9	0.8	56	31.3%	-1.60 [-1.85, -1.35]
Pinheiro de Oliveira 2010	5.522	6.88	20	3	3.44	20	3.7%	2.52 [-0.85, 5.89]
Simonis 2018	5.4	6.6	477	6	7.3	484	21.4%	-0.60 [-1.48, 0.28]
Yilmaz 2007	5.0536	3.7398	163	6.0528	3.7321	212	23.4%	-1.00 [-1.76, -0.24]
**Total (95% CI)**			**861**			**847**	**100.0%**	**-0.89 [-1.58, -0.20]**

Heterogeneity: Tau² = 0.38; Chi² = 14.56, df = 4 (P = 0.006); I² = 73%

Test for overall effect: Z = 2.53 (P = 0.01)

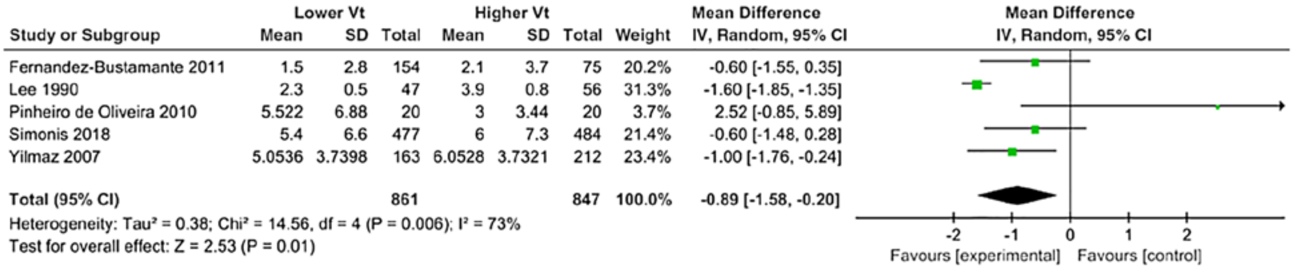

Figure S5. Incidence of pulmonary complications in patients without ARDS. ARDS, acute respiratory distress syndrome; M-H, Mantel-Haenszel.

Incidence of pulmonary complications in patients without ARDS available for lower versus higher tidal volume.

Study or Subgroup	Experimental Events	Experimental Total	Control Events	Control Total	Weight	Odds Ratio M-H, Fixed, 95% CI
Ge 2013	2	30	13	30	8.7%	0.09 [0.02, 0.47]
Severgnin 2013	1	27	7	26	4.9%	0.10 [0.01, 0.92]
Park 2016	3	21	9	19	5.8%	0.19 [0.04, 0.85]
Pi 2015	0	20	2	22	1.7%	0.20 [0.01, 4.43]
Kuzkov 2016	5	20	11	20	5.9%	0.27 [0.07, 1.04]
Shen 2015	7	60	18	60	11.4%	0.31 [0.12, 0.81]
Zamani	2	31	5	30	3.4%	0.34 [0.06, 1.94]
Futier 2013	35	200	72	200	42.7%	0.38 [0.24, 0.60]
Weingarten 2010	5	20	8	20	4.3%	0.50 [0.13, 1.93]
Soh 2018	10	39	10	39	5.3%	1.00 [0.36, 2.76]
Treschan 2012	13	50	11	51	5.8%	1.28 [0.51, 3.20]
**Total (95% CI)**		**518**		**517**	**100.0%**	**0.40 [0.30, 0.54]**
Total events	83		166			

Heterogeneity: $Chi^2 = 15.84$, $df = 10$ ($P = 0.10$); $I^2 = 37\%$

Test for overall effect: $Z = 5.98$ ($P < 0.00001$)

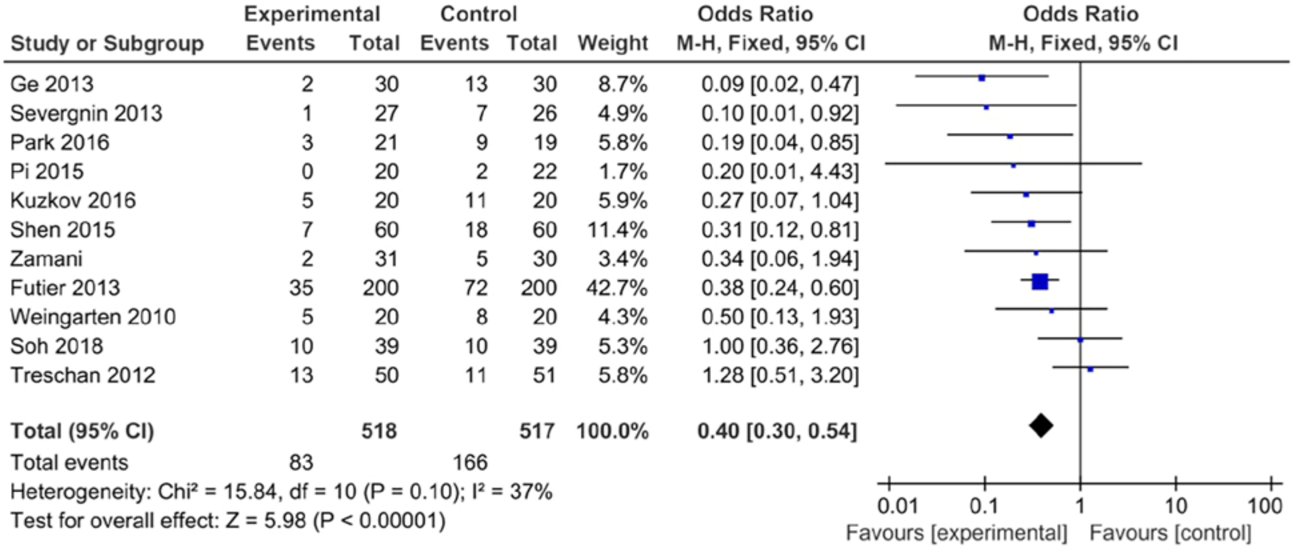

Figure S6. Hospital length of stay in patients without ARDS. ARDS, acute respiratory distress syndrome. CI, confidence intervals.

Hospital length of stay in patients without ARDS available for lower versus higher tidal volume. IV = Inverse variance.

	Lower Vt			Higher Vt				Mean Difference	Mean Difference
**Study or Subgroup**	**Mean**	**SD**	**Total**	**Mean**	**SD**	**Total**	**Weight**	**IV, Random, 95% CI**	**IV, Random, 95% CI**
Fernandez-Bustamante 2011	6.2	6.1	154	8.3	7.8	75	11.3%	-2.10 [-4.11, -0.09]	
Futier 2013	11.351	5.2271	200	13.702	8.9607	200	14.2%	-2.35 [-3.79, -0.91]	
Kuzkov 2016	29.0793	13.5634	20	39.122	20.744	20	0.9%	-10.04 [-20.90, 0.82]	
Park 2016	10	8	21	10	5	19	4.9%	0.00 [-4.09, 4.09]	
Pi 2015	19.439	6.3828	20	24.3709	8.7174	22	4.1%	-4.93 [-9.52, -0.34]	
Shen 2015	18.1	1.3	60	20.6	1.5	60	18.5%	-2.50 [-3.00, -2.00]	
Simonis 2018	20.4	23.8	475	21	21.1	481	8.0%	-0.60 [-3.45, 2.25]	
Soh 2018	8.065	2.309	39	8.355	3.8483	39	14.4%	-0.29 [-1.70, 1.12]	
Sundar 2011	5	1.5118	75	5.5	2.2682	74	18.1%	-0.50 [-1.12, 0.12]	
Treschan 2012	30	15	50	25	15	51	2.8%	5.00 [-0.85, 10.85]	
Weingarten 2010	7.5183	8.7763	20	11.9572	10.372	20	2.7%	-4.44 [-10.39, 1.52]	
**Total (95% CI)**			**1134**			**1061**	**100.0%**	**-1.49 [-2.54, -0.44]**	

Heterogeneity: Tau² = 1.49; Chi² = 40.12, df = 10 (P < 0.0001); I² = 75%

Test for overall effect: Z = 2.77 (P = 0.006)

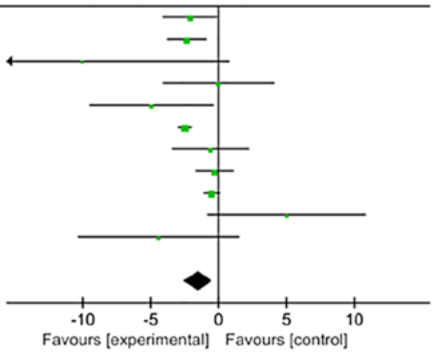

Supplement: Risk of bias for included randomized control trials. Red (-) indicates a high risk of bias; yellow (?) indicates unclear risk, and green (+) indicates a low risk of bias. [file Supplementary_Data1.pdf]
