# Supplementary material for: Association between tidal volume and mortality in patients without acute respiratory distress syndrome: A systematic review and meta-analysis
Source: Biomed Rep. 2026 Jun 23;25(2):98. doi: 10.3892/br.2026.2171 (PMC13320072; doi:10.3892/br.2026.2171)
Supplement: Newcastle-Ottawa quality assessment scale. [file Supplementary_Data2.pdf]

Table SI. Newcastle-Ottawa quality assessment scale.

Note: A study can be awarded a maximum of one star for each numbered item within the Selection and Outcome categories. A maximum of two stars can be given for Comparability

Selection

1) Representativeness of the exposed cohort
   a) truly representative of the average _______________ (describe) in the community
   b) somewhat representative of the average ______________ in the community
   c) selected group of users eg nurses, volunteers
   d) no description of the derivation of the cohort

2) Selection of the non exposed cohort
   a) drawn from the same community as the exposed cohort
   b) drawn from a different source
   c) no description of the derivation of the non exposed cohort

3) Ascertainment of exposure
   a) secure record (eg surgical records)
   b) structured interview
   c) written self report
   d) no description

4) Demonstration that outcome of interest was not present at start of study
   a) yes
   b) no

Comparability

1) Comparability of cohorts on the basis of the design or analysis
   a) study controls for ______AGE_______ (select the most important factor)
   b) study controls for any additional factor
   (This criteria could be modified to indicate specific control for a second important factor.)

Outcome

1) Assessment of outcome
   a) independent blind assessment
   b) record linkage
   c) self report
   d) no description

2) Was follow-up long enough for outcomes to occur
   a) yes (select an adequate follow up period for outcome of interest)
   b) no

3) Adequacy of follow up of cohorts
   a) complete follow up - all subjects accounted for
   b) subjects lost to follow up unlikely to introduce bias - small number lost - > ____ % (select an adequate %) follow up, or description provided of those lost)
   c) follow up rate < ____% (select an adequate %) and no description of those lost
   d) no statement

CODING MANUAL FOR COHORT STUDIES

SELECTION

1) Representativeness of the Exposed Cohort

Item is assessing the representativeness of exposed individuals in the community, not the representativeness of the sample of women from some general population. For example, subjects derived from groups likely to contain middle class, better educated, health oriented women are likely to be representative of postmenopausal estrogen users while they are not representative of all women (e.g. members of a health maintenance organisation (HMO) will be a representative sample of estrogen users. While the HMO may have an under-representation of ethnic groups, the poor, and poorly educated, these excluded groups are not the predominant users users of estrogen).

Allocation of stars as per rating sheet

2) Selection of the Non-Exposed Cohort

Allocation of stars as per rating sheet

3) Ascertainment of Exposure

Allocation of stars as per rating sheet

4) Demonstration That Outcome of Interest Was Not Present at Start of Study

In the case of mortality studies, outcome of interest is still the presence of a disease/ incident, rather than death. That is to say that a statement of no history of disease or incident earns a star.

COMPARABILITY

1) Comparability of Cohorts on the Basis of the Design or Analysis

A maximum of 2 stars can be allotted in this category

Either exposed and non-exposed individuals must be matched in the design and/or confounders must be adjusted for in the analysis. Statements of no differences between groups or that differences were not statistically significant are not sufficient for establishing comparability. Note: If the relative risk for the exposure of interest is adjusted for the confounders listed, then the groups will be considered to be comparable on each variable used in the adjustment.

There may be multiple ratings for this item for different categories of exposure (e.g. ever vs. never, current vs. previous or never)

Age = , Other controlled factors= ☆

OUTCOME

1) Assessment of Outcome

For some outcomes (e.g. fractured hip), reference to the medical record is sufficient to satisfy the requirement for confirmation of the fracture. This would not be adequate for vertebral fracture outcomes where reference to x-rays would be required.

a) Independent or blind assessment stated in the paper, or confirmation of the outcome by reference to secure records (x-rays, medical records, etc.) ☆
b) Record linkage (e.g. identified through ICD codes on database records) ☆
c) Self-report (i.e. no reference to original medical records or x-rays to confirm the outcome)
d) No description.

2) Was Follow-Up Long Enough for Outcomes to Occur

An acceptable length of time should be decided before quality assessment begins (e.g. 5 yrs. for exposure to breast implants)

3) Adequacy of Follow Up of Cohorts

This item assesses the follow-up of the exposed and non-exposed cohorts to ensure that losses are not related to either the exposure or the outcome.

Allocation of stars as per rating sheet

Table SII. Cross-sectional study quality assessment.

Item	Yes	No	Unclear
1) Define the source of information (survey, record review)	√		
2) List inclusion and exclusion criteria for exposed and unexposed subjects (cases and controls) or refer to previous publications	√		
3) Indicate time period used for identifying patients	√		
4) Indicate whether or not subjects were consecutive if not population-based			√
5) Indicate if evaluators of subjective components of study were masked to other aspects of the status of the participants			√
6) Describe any assessments undertaken for quality assurance purposes (e.g., test/retest of primary outcome measurements)			√
7) Explain any patient exclusions from analysis	√		
8) Describe how confounding was assessed and/or controlled.	√		
9) If applicable, explain how missing data were handled in the analysis	√		
10) Summarize patient response rates and completeness of data collection	√		
11) Clarify what follow-up, if any, was expected and the percentage of patients for which incomplete data or follow-up was obtained			√

An item would be scored '0' if it was answered 'NO' or 'UNCLEAR'; if it was answered 'YES', then the item scored '1'. Article quality was assessed as follows: Low quality = 0-3; moderate quality = 4-7; high quality = 8-11.

Table SIII. GRADE evidence profile for the studies in the meta-analysis.

Outcomes	Illustrative comparative risks[a] (95% CI)		Relative effect (95% CI)	No of Participants (studies)	Quality of the evidence (GRADE)
	Assumed risk	Corresponding risk			
	Control	28-30-ICU-hospital mortality			
28-30-ICU-hospital mortality	Study population		OR 0.78 (0.54 to 1.12)	2626 (10 studies)	⊕⊕⊝⊝ low[a,b]
	195 per 1000	159 per 1000 (116 to 213)			
	Moderate				
	50 per 1000	39 per 1000 (28 to 56)			
mortality subgroup - Phigh with difference	Study population		OR 0.5 (0.34 to 0.75)	1247 (6 studies)	⊕⊕⊕⊝ moderate[a]
	146 per 1000	79 per 1000 (55 to 114)			
	Moderate				
	50 per 1000	26 per 1000 (18 to 38)			
mortality subgroup - Phigh without difference	Study population		OR 1.11 (0.86 to 1.44)	1379 (4 studies)	⊕⊕⊕⊕ high
	238 per 1000	258 per 1000 (212 to 311)			
	Moderate				
	158 per 1000	172 per 1000 (139 to 213)			

[a]The basis for the assumed risk (e.g. the median control group risk across studies) is provided in footnotes. The corresponding risk (and its 95% CI) is based on the assumed risk in the comparison group and the relative effect of the intervention (and its 95% CI).

GRADE Working Group grades of evidence:

High quality: Further research is very unlikely to change our confidence in the estimate of effect.

Moderate quality: Further research is likely to have an important impact on our confidence in the estimate of effect and may change the estimate.

Low quality: Further research is very likely to have an important impact on our confidence in the estimate of effect and is likely to change the estimate. Very low quality: We are very uncertain about the estimate.

[a]exist bias, [b]not consist with each other, [c]the sample was small. CI: Confidence interval; OR: Odds ratio;
